# ﻿Redescription of the giant Southeast Asian millipede *Spirobolusmacrurus* Pocock, 1893 and its assignment to the new genus *Macrurobolus* gen. nov. (Diplopoda, Spirobolida, Pachybolidae)

**DOI:** 10.3897/zookeys.1087.71280

**Published:** 2022-02-22

**Authors:** Piyatida Pimvichai, Henrik Enghoff, Thierry Backeljau

**Affiliations:** 1 Department of Biology, Faculty of Science, Mahasarakham University, Mahasarakham 44150, Thailand Mahasarakham University Mahasarakham Thailand; 2 Natural History Museum of Denmark, University of Copenhagen, Universitetsparken 15, DK-2100 Copenhagen Ø, Denmark University of Copenhagen Copenhagen Ø Denmark; 3 Royal Belgian Institute of Natural Sciences, Vautierstraat 29, B-1000 Brussels, Belgium Royal Belgian Institute of Natural Sciences Brussels Belgium; 4 Evolutionary Ecology Group, University of Antwerp, Universiteitsplein 1, B-2610 Antwerp, Belgium University of Antwerp Antwerp Belgium

**Keywords:** Aphyly, Myanmar, taxonomy, Thailand

## Abstract

A new genus of the millipede family Pachybolidae from Southeast Asia is described: *Macrurobolus***gen. nov.**, with *Spirobolusmacrurus* Pocock, 1893 as type species. This latter species is DNA barcoded (COI) and redescribed based on male morphological characters, which hitherto were unknown. The new genus differs from other pachybolid genera by having (1) the preanal ring process long and protruding beyond the anal valves and (2) the anterior gonopod telopodite distally abruptly narrowed, forming an extremely long, slender, elevated process curved caudad. Given that *Macrurobolus***gen. nov.** is a monotypic genus, it is aphyletic and thus requires further taxonomic revision.

## ﻿Introduction

*Spirobolusmacrurus* Pocock, 1893 is, with its length of up to 110 mm and diameter of up to 10 mm, the largest pachybolid millipede in SE Asia, but despite its large size, the species is still poorly known. Its original description was based on a single female specimen from Kawkareet, Tenasserim, Myanmar, and did not include the genital parts. Yet, Pocock (1893) separated *S.macrurus* from other *Spirobolus* species by its much longer and thinner preanal ring process. Much later, Hoffman (1962: 773) transferred the species to the genus *Tonkinbolus* Verhoeff, 1938 and remarked “said to be closely related to *moulmeinensis*, differing only in the longer and more slender epiproct”. However, based on gonopod characters and strongly supported by DNA sequence data, Pimvichai et al. (2018) assigned *Tonkinbolusscaber* Verhoeff, 1938 (type species of *Tonkinbolus*) to the genus *Litostrophus* Chamberlin, 1921. Thus, *Tonkinbolus* became a subjective junior synonym of *Litostrophus*. At the same time, Pimvichai et al. (2018) moved all other *Tonkinbolus* species, including *T.macrurus*, to the genus *Atopochetus* Attems, 1953 because they share the unique anterior gonopod telopodite of this genus. Yet, since *T.macrurus* was until then only characterised on the basis of a single female specimen, its transfer to *Atopochetus* was qualified as “incertae sedis” (Pimvichai et al. 2018).

In the present paper we redescribe and barcode *Spirobolusmacrurus* based on an old male specimen discovered in the collections of the Natural History Museum of Denmark, Copenhagen, and new live material, including an adult male specimen, collected during recent fieldwork in Thailand. As a result we also create the new genus *Macrurobolous* gen. nov. to accommodate *Spirobolusmacrurus*, so that this species will be referred to as *Macrurobolusmacrurus* comb. nov.

## ﻿Material and methods

Live specimens were hand collected and preserved in 70% ethanol for morphological study or placed in a freezer at –20 °C for DNA analysis. Specimens were also examined from the following collections:

**CUMZ**Museum of Zoology, Chulalongkorn University, Bangkok, Thailand;

**NHMD**Natural History Museum of Denmark, University of Copenhagen, Denmark.

This research was conducted under the approval of the Animal Care and Use regulations (numbers U1-07304-2560 and IACUC-MSU-037/2019) of the Thai government.

### ﻿Morphology

Gonopods were photographed with a digital camera manipulated via the program Helicon Remote (v. 3.1.1.w). The Zerene Stacker Pro software was used for image-stacking. Drawings were made using a stereomicroscope. Samples for scanning electron microscopy (SEM) were air-dried directly from alcohol and sputter-coated for 250 s with gold. SEM micrographs were taken with an environmental scanning electron microscope (ESEM)-FEI Quanta 200. Voucher specimens were deposited in the collections of CUMZ and NHMD.

### ﻿DNA extraction, amplification, and sequencing

Total genomic DNA was extracted from legs of a male specimen of *Macrurobolusmacrurus*, comb. nov. from Wat Tham Inthanin, Mae Sot District, Tak Province, Thailand (CUMZ-D00147) using the NucleoSpin Tissue kit (Macherey-Nagel, Düren, Germany) following the manufacturer’s instructions. PCR amplifications and sequencing of the standard mitochondrial COI DNA barcoding fragment (Hebert et al. 2003) were done as described by Pimvichai et al. (2020). The COI fragment was amplified with the primers LCO-1490 and HCO-2198 (Folmer et al. 1994). The new COI nucleotide sequence has been deposited in GenBank under accession number MZ905519. Sample data and voucher codes are provided in Table 1.

**Table 1.** Specimens from which the COI gene fragment was sequenced. CUMZ, Museum of Zoology, Chulalongkorn University, Bangkok, Thailand; NHMD, Natural History Museum of Denmark; NHMW, Naturhistorisches Museum, Vienna, Austria; NHM, The Natural History Museum, London, United Kingdom. Names of countries are in capitals. Abbreviations after species names refer to the isolate of each sequence. GenBank accession numbers are indicated for each species.

	Voucher code	Locality	COI
**Genus *Apeuthes***			
*A.maculatus* Amc	NHMW-Inv. No.2395	South Annam, Vietnam	MF187404
*A.maculatus* Am26	NHMD-621697	Nha Trang, Bao Dai Villas Hotel, in garden, Vietnam	MZ567159
*A.fimbriatus* BMP	CUMZ-D00144	Bach Ma Peak, Da Nang, Vietnam	MZ567160
*A.longeligulatus* TPP	CUMZ-D00140	Tham Phet Po Thong, Klong Hard, Sa Kaeo, Thailand	MZ567161
*A.pollex* SMR	CUMZ-D00141	Sra Morakot, Klongthom, Krabi, Thailand	MZ567162
*A.pollex* SML	CUMZ-D00142	Koh 8, Similan islands, Phang-Nga, Thailand	MZ567163
*A.pollex* WTS	CUMZ-D00143	Tham Sue Temple, Muang, Krabi, Thailand	MZ567164
?*A.spininavis* ABB	CUMZ-D00145	Air Banun, Perak, Malaysia	MZ567165
**Genus *Atopochetus***			
*A.anaticeps* SVL	CUMZ-D00091	Srivilai temple, Chalermprakiet, Saraburi, Thailand	MF187405
*A.dollfusii* DOL	NHM	Cochinchina, Vietnam	MF187412
*A.helix* SPT	CUMZ-D00094	Suan Pa Thong Pha Phum, Kanchanaburi, Thailand	MF187416
*A.moulmeinensis* TAK	CUMZ-D00095	km 87, Tha Song Yang, Tak, Thailand	MF187417
*A.setiferus* HPT	CUMZ-D00097	Hub Pa Tard, Lan-Sak, Uthaithani, Thailand	MF187419
*A.spinimargo* Ton27	NHMD-00047013	Koh Yo, Songkhla, Thailand	MF187423
*A.truncatus* SML	CUMZ-D00101	Koh 8, Similan islands, Phang-Nga, Thailand	MF187424
*A.uncinatus* KMR	CUMZ-D00102	Khao Mar Rong, Bangsapan, Prachuapkhirikhan, Thailand	MF187425
*A.weseneri* Tos29	NHMD-00047003	Supar Royal Beach Hotel, Khanom, Nakhonsrithammarat, Thailand	MF187431
**Genus *Aulacobolus***			
*A.uncopygus* Auc	NHMW-Inv. No.2375	Nilgiris, South India, India	MF187433
**Genus *Benoitolus***			
*B.birgitae* BBG	NHMD 621687	Chiang Dao, Chiang-Mai, Thailand	MT328992
**Genus *Coxobolellus***			
*C.albiceps* Stpw	CUMZ-D00121	Tham Pha Tub, Muang District, Nan Province, Thailand (green individual)	MT328994
*C.compactogonus* SKR	CUMZ-D00134	Sakaerat Environmental Research Station, Wang Nam Khiao District, Nakhon Ratchasima Province, Thailand	MT328998
*C.fuscus* HKK	CUMZ-D00133	Kroeng Krawia waterfall, Sangkhla Buri District, Kanchanaburi Province, Thailand	MT328999
*C.nodosus* SPW	CUMZ-D00126	Chao Por Phawo Shrine, Mae Sot District, Tak Province, Thailand	MT329000
*C.serratus* KKL	CUMZ-D00132	Khao Kalok, Pran Buri District, Prachuap Khiri Khan Province, Thailand	MT329001
*C.simplex* TNP	CUMZ-D00136	Tham Pha Pha Ngam, Mae Prik District, Lampang Province, Thailand	MT329002
*C.tenebris* TPL	CUMZ-D00120	Wat Tham Phrom Lok Khao Yai, Sai Yok District, Kanchanaburi Province, Thailand	MT329004
*C.tigris* TYE	CUMZ-D00131	Tham Yai I, Pathio District, Chumphon Province, Thailand	MT329006
*C.transversalis* Stpg	CUMZ-D00125	Tham Pha Tub, Muang District, Nan Province, Thailand	MT329007
*C.valvatus* BRC	CUMZ-D00128	Tham Borichinda, Chom Thong District, Chiang-Mai Province, Thailand	MT329008
**Genus *Leptogoniulus***			
*L.sorornus* BTN	CUMZ-D00109	Botanical Garden, Penang, Malaysia	MF187434
**Genus *Litostrophus***			
*L.chamaeleon* PPT	CUMZ-D00111	Phu Pha terb, Mukdahan, Thailand	MF187436
*L.saraburensis* PKS	CUMZ-D00113	Phukhae Botanical Garden, Saraburi, Thailand	MF187438
*L.segregatus* Ls19	NHMD 621686	Koh Kut, Trad, Thailand	MF187440
**Genus *Macrurobolus* gen. nov.**			
*M.macrurus* comb. nov.	CUMZ- D00147	Wat Tham Inthanin, Mae Sot District, Tak Province, Thailand	MZ905519
**Genus *Madabolus***			
*M.maximus* Mm4	NHMD-00047007	de Toliara Province, Parc National de Bermaraha, South Bank of Manambolo River, Near Tombeau Vazimba, Madagascar	MF187441
**Genus *Narceus***			
*N.annularis*			NC_003343.1
**Genus *Parabolus***			
*P.dimorphus* Pd34	NHMD-00047004	Dar es Salaam, Tanzania	MF187442
**Genus *Paraspirobolus***			
*P.lucifugus*			AB608779.1
**Genus *Pelmatojulus***			
*P.tigrinus* Pt2	NHMD-00047008	Southern part of the Comoé N.P., 30 km north of Kakpin, Côte d’Ivoire	MF187443
*P.togoensis* Pto6	NHMD-00047006	Biakpa, Ghana	MF187444
**Genus *Pseudospirobolellus***			
*P.avernus* GPG	CUMZ-D00117	Gua Pulai, Gua Musang, Kelantan, Malaysia	MT329011
*Pseudospirobolellus* sp. KCS	CUMZ-D00118	Koh Chuang, Sattahip, Chonburi, Thailand	MT329012
**Genus *Rhinocricus***			
*R.parcus* Rp49	NHMD-00047009	Puerto Rico, Usa	MF187449
***Genus Trachelomegalus***			
*Trachelomgalus* sp. Tr54	NHMD-00047012	Borneo Sabah, Malaysia	MF187445
**Genus *Trigoniulus***			
*T.corallinus* Tco15	NHMD-00047010	Vientiane, Laos	MF187446
**Outgroup**			
**Genus *Anurostreptus***			
*A.barthelemyae* Tlb	CUMZ-D00003	Thale-Ban N.P., Khuan-Don, Satun, Thailand	KC519469
**Genus *Chonecambala***			
*C.crassicauda* Ttp	CUMZ-D00001	Ton-Tong waterfall, Pua, Nan, Thailand	KC519467
**Genus *Thyropygus***			
*T.allevatus* Bb	CUMZ-D00013	BangBan, Ayutthaya, Thailand	KC519479

### ﻿Alignment and phylogenetic analysis

The COI data included 48 specimens, representing 17 genera and 40 nominal species of ingroup taxa (Table 1). Three species of the order Spirostreptida, viz. *Anurostreptusbarthelemyae* Demange, 1961 (Harpagophoridae), *Chonecambalacrassicauda* Mauriès & Enghoff, 1990 (Pericambalidae), and *Thyropygusallevatus* (Karsch, 1881) (Harpagophoridae) were used as outgroup.

CodonCode Aligner (v. 4.0.4, CodonCode Corporation) was used to assemble the forward and reverse sequences and to check for errors and ambiguities. Sequences were checked with the Basic Local Alignment Search Tool (BLAST) provided by NCBI and compared with reference sequences in GenBank. Next, sequences were aligned using MUSCLE (v. 3.6, see http://www.drive5.com/muscle; Edgar 2004). The COI alignments consisted of 660 bp. The sequences were checked for ambiguous nucleotide sites, saturation and phylogenetic signal using DAMBE (v. 5.2.65. see http://www.dambe.bio.uottawa.ca/DAMBE/dambe.aspx; Xia 2018). MEGA (v. X, see http://www.megasoftware.net; Kumar et al. 2018) was used to (1) check for stop codons, (2) translate COI protein-coding sequences into amino acids, and (3) calculate uncorrected pairwise *p*-distances among sequences.

Phylogenetic trees were constructed using maximum likelihood (ML), Bayesian inference (BI), and neighbor-joining (NJ). The shape parameter of the gamma distribution, based on 16 rate categories, was estimated using maximum-likelihood analysis. ML trees were inferred with RAxML (v. 8.2.12, see http://www.phylo.org/index.php/tools/raxmlhpc2_tgb.html; Stamatakis 2014) through the CIPRES Science Gateway (Miller et al. 2010) using a GTR+G substitution model and 1000 bootstrap replicates to assess branch support. BI trees were constructed with MrBayes (v. 3.2.7a, see http://www.phylo.org/index.php/tools/mrbayes_xsede.html; Huelsenbeck and Ronquist 2001). Substitution models were inferred using PartitionFinder 2 on XSEDE (v. 2.1.1, see http://www.phylo.org//index.php/tools/partitionfinder2_xsede.html; Lanfear et al. 2017) through the CIPRES Science Gateway (Miller et al. 2010). BI trees were run for 2 million generations (heating parameter was 0.05), sampling every 1000 generations. Convergences were confirmed by verifying that the standard deviations of split frequencies were below 0.01. Then the first 1000 trees were discarded as burn-in, so that the final consensus tree was built from the last 3002 trees. Support for nodes was assessed by posterior probabilities. NJ trees were constructed with MEGA v. X using the Kimura 2-parameter model and 1000 bootstrap replicates.

For ML and NJ trees we consider branches with bootstrap values (BV) of ≥ 70% to be well supported (Hillis and Bull 1993) and < 70% as poorly supported. For BI trees, we consider branches with posterior probabilities (PP) of ≥ 0.95 to be well supported (San Mauro and Agorreta 2010) and below as poorly supported.

## ﻿Results

The uncorrected *p*-distance between the sequences ranged from 0.03 to 0.25 (Tables 2, 3). The mean interspecific sequence divergence within *Atopochetus* was 0.13 (range: 0.08–0.16). The mean sequence divergence between *Atopochetus* and *M.macrurus* comb. nov. was 0.15 (range: 0.14–0.17). The mean interspecific sequence divergence within *Litostrophus* was 0.10 (range: 0.09–0.11). The mean sequence divergence between *Litostrophus* and *M.macrurus* comb. nov. was 0.13 (range: 0.11–0.14).

**Table 2.** Estimates of COI sequence divergences (uncorrected *p*-distances) within and among Pachybolidae species and related taxa (rounded to two decimals).

**1**	***Macrurobolusmacrurus* comb. nov.**	1	2	3	4	5	6	7	8	9	10	11	12	13	14	15	16	17	18	19	20	21	22	23	24	25	26	27
2	*Apeutheslongeligulatus* TPP	0.18																										
3	?*Apeuthesspininavis* ABB	0.18	0.14																									
4	*Apeuthesfimbriatus* BMP1	0.21	0.15	0.16																								
5	*Apeuthespollex* SML	0.18	0.14	0.15	0.15																							
6	*Apeuthespollex* SMR	0.18	0.14	0.14	0.15	0.06																						
7	*Apeuthespollex* WTS	0.18	0.15	0.15	0.15	0.04	0.07																					
8	*Apeuthesmaculatus* Amc	0.17	0.11	0.12	0.14	0.11	0.11	0.11																				
9	*Apeuthesmaculatus* Am26	0.18	0.13	0.14	0.15	0.13	0.13	0.13	0.03																			
10	*Atopochetusanaticeps* SVL	0.16	0.20	0.19	0.23	0.18	0.18	0.19	0.19	0.20																		
11	*Atopochetusdollfusii* DOL	0.14	0.19	0.20	0.22	0.19	0.19	0.20	0.20	0.21	0.11																	
12	*Atopochetushelix* SPT	0.15	0.23	0.19	0.22	0.20	0.21	0.20	0.21	0.22	0.14	0.13																
13	*Atopochetusmoulmeinensis* TAK	0.17	0.22	0.22	0.23	0.22	0.23	0.23	0.22	0.23	0.14	0.12	0.15															
14	*Atopochetussetiferus* HPT	0.14	0.20	0.20	0.22	0.18	0.18	0.19	0.19	0.20	0.08	0.09	0.14	0.13														
15	*Atopochetusspinimargo* Ton27	0.17	0.22	0.22	0.22	0.21	0.20	0.20	0.22	0.22	0.15	0.14	0.14	0.16	0.14													
16	*Atopochetustruncatus* SML	0.15	0.20	0.20	0.22	0.20	0.19	0.21	0.19	0.21	0.13	0.10	0.12	0.14	0.12	0.14												
17	*Atopochetusuncinatus* KMR	0.16	0.21	0.20	0.21	0.19	0.20	0.20	0.20	0.22	0.13	0.14	0.14	0.15	0.13	0.15	0.13											
18	*Atopochetusweseneri* Tos29	0.16	0.21	0.20	0.21	0.21	0.20	0.22	0.20	0.21	0.14	0.12	0.14	0.13	0.12	0.16	0.10	0.13										
19	*Aulacobolusuncopygus* Auc	0.17	0.17	0.18	0.20	0.16	0.17	0.17	0.17	0.18	0.18	0.18	0.20	0.21	0.19	0.22	0.19	0.20	0.22									
20	*Coxobolellusalbiceps* Stpw	0.21	0.18	0.21	0.20	0.18	0.17	0.18	0.18	0.19	0.20	0.22	0.22	0.24	0.22	0.23	0.21	0.21	0.22	0.18								
21	*Coxobolelluscompactogonus* SKR	0.23	0.18	0.19	0.21	0.19	0.18	0.19	0.19	0.21	0.21	0.21	0.22	0.24	0.21	0.23	0.21	0.21	0.22	0.19	0.14							
22	*Coxobolellusfuscus* HKK	0.22	0.19	0.20	0.20	0.17	0.18	0.18	0.20	0.21	0.20	0.22	0.22	0.24	0.20	0.23	0.23	0.20	0.23	0.19	0.12	0.13						
23	*Coxobolellusnodosus* SPW	0.21	0.18	0.20	0.22	0.18	0.19	0.19	0.20	0.20	0.21	0.20	0.21	0.24	0.21	0.23	0.22	0.22	0.23	0.18	0.11	0.13	0.11					
24	*Coxobolellusserratus* KKL	0.21	0.18	0.20	0.20	0.18	0.18	0.18	0.18	0.20	0.20	0.21	0.22	0.23	0.20	0.23	0.21	0.22	0.22	0.19	0.13	0.14	0.12	0.13				
25	*Coxobolellussimplex* TNP	0.20	0.18	0.18	0.20	0.18	0.18	0.18	0.19	0.20	0.21	0.22	0.21	0.23	0.22	0.23	0.23	0.23	0.22	0.20	0.13	0.12	0.12	0.12	0.11			
26	*Coxobolellustenebris* TPL	0.22	0.19	0.18	0.21	0.18	0.18	0.18	0.18	0.20	0.21	0.22	0.23	0.25	0.22	0.24	0.23	0.23	0.23	0.19	0.13	0.10	0.12	0.12	0.14	0.11		
27	*Coxobolellustigris* TYE	0.23	0.19	0.21	0.22	0.20	0.20	0.20	0.20	0.20	0.19	0.22	0.22	0.25	0.21	0.24	0.22	0.22	0.22	0.21	0.13	0.14	0.12	0.13	0.12	0.13	0.15	
28	*Coxobolellustransversalis* Stpg	0.21	0.18	0.20	0.21	0.18	0.18	0.19	0.18	0.19	0.20	0.20	0.20	0.23	0.21	0.21	0.20	0.22	0.22	0.18	0.08	0.15	0.12	0.11	0.12	0.12	0.14	0.13
29	*Coxobolellusvalvatus* BRC	0.21	0.17	0.19	0.20	0.16	0.16	0.17	0.17	0.18	0.20	0.21	0.20	0.24	0.20	0.23	0.22	0.22	0.22	0.17	0.10	0.13	0.11	0.07	0.13	0.12	0.12	0.13
*30*	*Paraspiroboluslucifugus*	0.25	0.25	0.22	0.23	0.22	0.22	0.22	0.22	0.23	0.23	0.23	0.23	0.23	0.23	0.23	0.23	0.23	0.24	0.24	0.24	0.23	0.23	0.24	0.25	0.25	0.24	0.24
31	*Leptogoniulussorornus* BTN	0.18	0.16	0.14	0.16	0.14	0.14	0.15	0.13	0.14	0.18	0.18	0.19	0.21	0.19	0.20	0.19	0.22	0.22	0.17	0.21	0.20	0.20	0.19	0.19	0.18	0.20	0.20
32	*Litostrophuschamaeleon* PPT	0.14	0.20	0.19	0.20	0.18	0.18	0.20	0.18	0.19	0.17	0.16	0.15	0.18	0.16	0.18	0.15	0.17	0.17	0.19	0.20	0.21	0.20	0.20	0.20	0.21	0.21	0.20
33	*Litostrophussaraburensis* PKS	0.11	0.18	0.18	0.20	0.18	0.17	0.18	0.16	0.17	0.16	0.15	0.15	0.17	0.15	0.16	0.14	0.16	0.18	0.18	0.18	0.20	0.19	0.19	0.20	0.20	0.20	0.20
34	*Litostrophussegregatus* Ls19	0.13	0.19	0.19	0.20	0.18	0.18	0.19	0.18	0.20	0.13	0.13	0.15	0.16	0.13	0.15	0.14	0.14	0.17	0.18	0.21	0.21	0.21	0.21	0.20	0.22	0.21	0.21
35	*Madabolusmaximus* Mm4	0.19	0.20	0.18	0.20	0.19	0.19	0.20	0.20	0.21	0.21	0.20	0.19	0.22	0.22	0.21	0.20	0.20	0.22	0.18	0.20	0.23	0.22	0.21	0.22	0.22	0.24	0.22
*36*	*Narceusannularis*	0.20	0.21	0.20	0.20	0.21	0.21	0.21	0.21	0.22	0.23	0.20	0.21	0.22	0.21	0.20	0.20	0.21	0.21	0.20	0.22	0.23	0.21	0.23	0.22	0.22	0.23	0.22
37	*Parabolusdimorphus* Pd34	0.20	0.21	0.21	0.22	0.19	0.19	0.19	0.20	0.21	0.18	0.20	0.19	0.22	0.18	0.20	0.21	0.19	0.21	0.19	0.18	0.22	0.19	0.18	0.20	0.20	0.20	0.17
38	*Pelmatojulustigrinus* Pt2	0.18	0.18	0.18	0.19	0.18	0.17	0.19	0.17	0.18	0.22	0.22	0.20	0.23	0.22	0.23	0.22	0.22	0.22	0.16	0.20	0.20	0.20	0.21	0.22	0.22	0.22	0.20
39	*Pelmatojulustogoensis* Pto6	0.21	0.19	0.20	0.18	0.18	0.17	0.17	0.18	0.20	0.21	0.22	0.22	0.22	0.20	0.20	0.21	0.20	0.21	0.17	0.19	0.20	0.20	0.19	0.19	0.21	0.20	0.20
40	*Pseudospirobolellusavernus* GPG	0.21	0.21	0.19	0.23	0.19	0.20	0.20	0.20	0.22	0.21	0.22	0.22	0.23	0.21	0.23	0.22	0.23	0.23	0.20	0.20	0.21	0.20	0.21	0.20	0.21	0.21	0.20
41	*Pseudospirobolellus* sp. KCS	0.23	0.22	0.22	0.22	0.22	0.22	0.21	0.23	0.23	0.23	0.23	0.21	0.23	0.23	0.22	0.22	0.23	0.23	0.22	0.22	0.22	0.21	0.21	0.22	0.22	0.23	0.22
42	*Rhinocricusparcus* Rp49	0.24	0.24	0.23	0.23	0.23	0.23	0.22	0.23	0.24	0.24	0.21	0.22	0.22	0.24	0.21	0.23	0.22	0.23	0.22	0.25	0.25	0.25	0.25	0.25	0.25	0.25	0.24
43	*Trachelomegalus* sp. Tr54	0.19	0.20	0.19	0.20	0.19	0.19	0.20	0.20	0.22	0.19	0.18	0.17	0.20	0.19	0.18	0.18	0.18	0.18	0.20	0.21	0.22	0.24	0.23	0.22	0.22	0.23	0.24
44	*Trigoniuluscorallinus* Tco15	0.18	0.15	0.14	0.13	0.13	0.13	0.14	0.12	0.12	0.18	0.19	0.20	0.23	0.19	0.20	0.20	0.21	0.21	0.17	0.18	0.18	0.17	0.18	0.19	0.17	0.18	0.17
45	*Anurostreptusbarthelemyae* Tlb	0.23	0.21	0.21	0.22	0.20	0.20	0.19	0.21	0.22	0.22	0.22	0.23	0.24	0.23	0.22	0.24	0.23	0.24	0.20	0.19	0.21	0.19	0.19	0.20	0.19	0.20	0.19
*46*	*Chonecambalacrassicauda*	0.24	0.23	0.22	0.21	0.22	0.22	0.21	0.21	0.23	0.24	0.24	0.24	0.23	0.24	0.23	0.24	0.22	0.24	0.22	0.23	0.23	0.22	0.23	0.22	0.22	0.23	0.22
47	*Thyropygusallevatus* Bb	0.21	0.21	0.21	0.21	0.20	0.21	0.20	0.21	0.22	0.21	0.21	0.22	0.23	0.23	0.23	0.22	0.21	0.24	0.20	0.20	0.19	0.20	0.20	0.19	0.19	0.20	0.19

**Table 2.** Continued.

**1**	***Macrurobolusmacrurus* comb. nov.**	28	29	30	31	32	33	34	35	36	37	38	39	40	41	42	43	44	45	46
2	*Apeutheslongeligulatus* TPP																			
3	?*Apeuthesspininavis* ABB																			
4	*Apeuthesfimbriatus* BMP1																			
5	*Apeuthespollex* SML																			
6	*Apeuthespollex* SMR																			
7	*Apeuthespollex* WTS																			
8	*Apeuthesmaculatus* Amc																			
9	*Apeuthesmaculatus* Am26																			
10	*Atopochetusanaticeps* SVL																			
11	*Atopochetusdollfusii* DOL																			
12	*Atopochetushelix* SPT																			
13	*Atopochetusmoulmeinensis* TAK																			
14	*Atopochetussetiferus* HPT																			
15	*Atopochetusspinimargo* Ton27																			
16	*Atopochetustruncatus* SML																			
17	*Atopochetusuncinatus* KMR																			
18	*Atopochetusweseneri* Tos29																			
19	*Aulacobolusuncopygus* Auc																			
20	*Coxobolellusalbiceps* Stpw																			
21	*Coxobolelluscompactogonus* SKR																			
22	*Coxobolellusfuscus* HKK																			
23	*Coxobolellusnodosus* SPW																			
24	*Coxobolellusserratus* KKL																			
25	*Coxobolellussimplex* TNP																			
26	*Coxobolellustenebris* TPL																			
27	*Coxobolellustigris* TYE																			
28	*Coxobolellustransversalis* Stpg																			
29	*Coxobolellusvalvatus* BRC	0.11																		
*30*	*Paraspiroboluslucifugus*	0.24	0.23																	
31	*Leptogoniulussorornus* BTN	0.19	0.19	0.24																
32	*Litostrophuschamaeleon* PPT	0.21	0.20	0.24	0.18															
33	*Litostrophussaraburensis* PKS	0.18	0.19	0.24	0.18	0.11														
34	*Litostrophussegregatus* Ls19	0.20	0.20	0.25	0.18	0.11	0.09													
35	*Madabolusmaximus* Mm4	0.21	0.20	0.24	0.20	0.19	0.18	0.20												
*36*	*Narceusannularis*	0.22	0.22	0.21	0.21	0.20	0.20	0.21	0.20											
37	*Parabolusdimorphus* Pd34	0.19	0.18	0.25	0.21	0.19	0.18	0.20	0.17	0.20										
38	*Pelmatojulustigrinus* Pt2	0.21	0.19	0.24	0.19	0.20	0.20	0.20	0.18	0.19	0.19									
39	*Pelmatojulustogoensis* Pto6	0.20	0.18	0.25	0.18	0.18	0.18	0.19	0.19	0.20	0.18	0.17								
40	*Pseudospirobolellusavernus* GPG	0.20	0.21	0.22	0.19	0.23	0.21	0.21	0.21	0.22	0.23	0.20	0.21							
41	*Pseudospirobolellus* sp. KCS	0.22	0.22	0.22	0.21	0.23	0.22	0.22	0.24	0.22	0.21	0.22	0.22	0.14						
42	*Rhinocricusparcus* Rp49	0.25	0.24	0.22	0.22	0.22	0.23	0.23	0.22	0.20	0.23	0.21	0.22	0.22	0.21					
43	*Trachelomegalus* sp. Tr54	0.22	0.23	0.24	0.21	0.18	0.17	0.15	0.21	0.21	0.21	0.19	0.21	0.22	0.20	0.22				
44	*Trigoniuluscorallinus* Tco15	0.17	0.16	0.23	0.14	0.18	0.16	0.17	0.18	0.20	0.19	0.18	0.17	0.23	0.22	0.23	0.20			
45	*Anurostreptusbarthelemyae* Tlb	0.19	0.18	0.23	0.22	0.21	0.20	0.22	0.22	0.21	0.21	0.21	0.20	0.22	0.21	0.23	0.23	0.19		
*46*	*Chonecambalacrassicauda*	0.22	0.21	0.23	0.21	0.23	0.22	0.24	0.24	0.23	0.21	0.22	0.24	0.23	0.23	0.23	0.22	0.22	0.19	
47	*Thyropygusallevatus* Bb	0.20	0.19	0.22	0.20	0.21	0.20	0.22	0.21	0.20	0.21	0.23	0.21	0.22	0.22	0.21	0.24	0.20	0.15	0.20

PartitionFinder indicated that the best substitution model for BI analysis was GTR+ G. The ML, BI, and NJ trees were congruent with respect to some of the well-supported branches (by visual inspection of the branching pattern). Yet, in several instances BI provided good support for branches that were not well-supported by both ML and NJ (e.g., the *Litostrophus* + *Benoitolus* clade or the *Coxobolellus* + *Pseudospirobolellus* clade).

**Figure 1.**
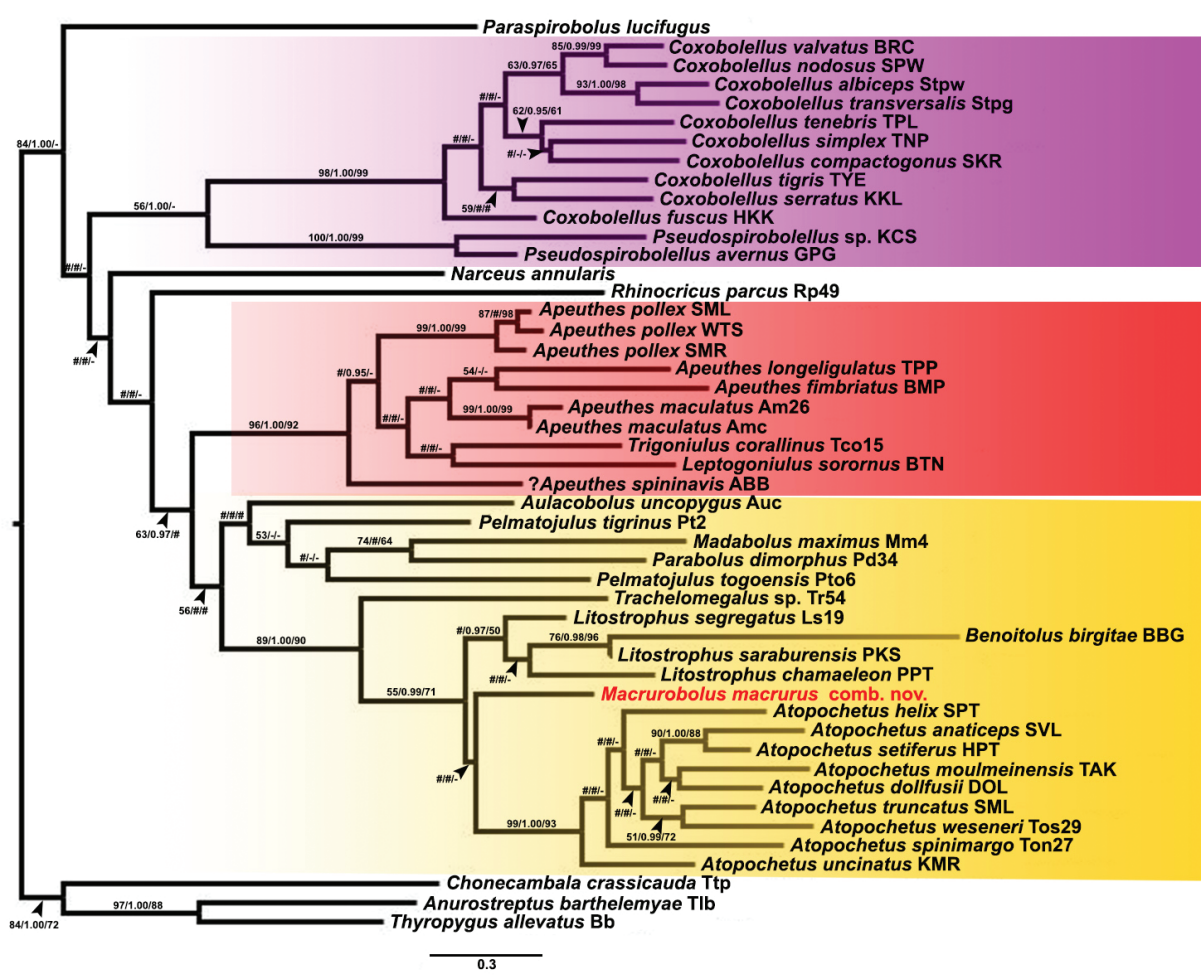
Phylogenetic relationships of pachybolid and several other spirobolidan millipede species based on maximum likelihood analysis (ML) of a 660 bp COI gene fragment. Numbers at nodes indicate branch support based on bootstrapping (ML) / posterior probabilities (BI) / bootstrapping (NJ). Scale bar: 0.3 substitutions/site. # indicates branches with < 50% ML and NJ bootstrap support or < 0.95 posterior probability, - indicates non-supported branches. The coloured areas mark the Pseudospirobolellidae (minus *Benoitolus*) (purple), Trigoniulinae (red), and non-trigoniuline Pachybolidae (plus *Benoitolus*) (yellow).

In the phylogenetic trees (Fig. 1) the clade of Pachybolidae + *Benoitolus* is poorly supported by ML (BV = 63) and NJ (BV = 27), but well supported by BI (PP = 0.97), while Trigoniulinae is well supported by the three methods (BV = 96 and 92; PP = 1.00). Although the monophyly of Pachybolidae is clearly challenged by the inclusion of *Benoitolus*, which involves a long branch, removing *Benoitolus* from the analysis yields a Pachybolidae clade with the same pattern of support as the Pachybolidae + *Benoitolus* clade (Suppl. material 1).

Irrespective of the in- or exclusion of *Benoitolus*, *Macrurobolusmacrurus* comb. nov. is nested within a clade comprising *Litostrophus* and *Atopochetus*. Yet, this clade is poorly supported by ML, well supported (but just so) by NJ, and convincingly well supported by BI. The position of *M.macrurus* comb. nov. within this clade, however, is poorly supported by the three methods.

### ﻿Taxonomy

#### Class DIPLOPODA de Blainville in Gervais, 1844

**Order SPIROBOLIDA Bollman, 1893**

**Suborder TRIGONIULIDEA Attems, 1909**

##### Family PACHYBOLIDAE Cook, 1897

###### ﻿Genus Macrurobolus gen. nov.

Taxon classificationAnimaliaSpirobolidaPachybolidae

9DB041FE-8530-5BD7-8CB8-C3F40FA5F9A4

http://zoobank.org/A428FDFE-D777-4B7B-8D29-F603088A0AC2

**Figure 2.**
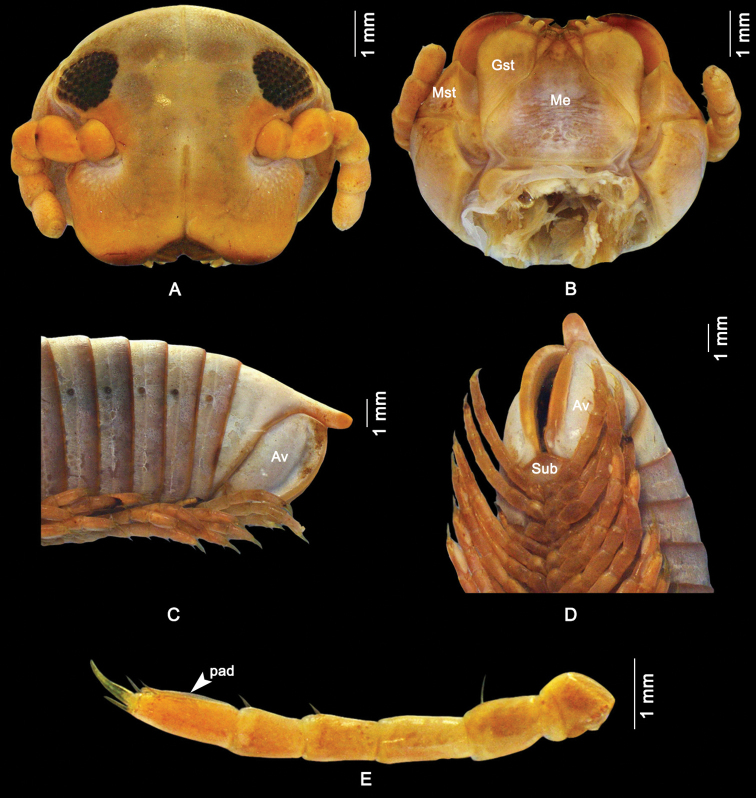
External morphology of a male *Macrurobolusmacrurus* comb. nov. from Wat Tham Inthanin, Thailand, CUMZ-D00147-1 **A** head, frontal view **B** gnathochilarium, ventral view **C** posterior end, lateral view **D** posterior end, latero-ventral view **E** midbody leg, latero-ventral view. Av = anal valves; Gst = gnathochilarial stipes; Me = mentum; Mst = mandibular stipes; Sub = subanal scale.

**Figure 3.**
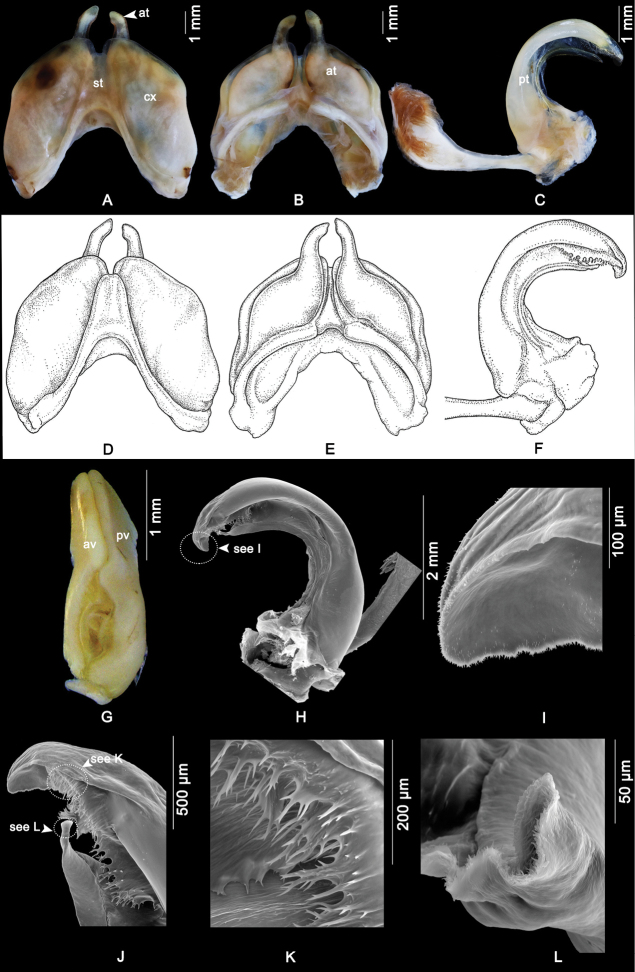
Male (A–F, H–L) and female (G) genital parts of *Macrurobolusmacrurus* comb. nov. (specimens from Wat Tham Inthanin, Thailand, CUMZ-D00147-1) **A** anterior gonopod, anterior view **B** anterior gonopod, posterior view **C** right posterior gonopod, posterior-mesal view **D** anterior gonopod, anterior view **E** anterior gonopod, posterior view **F** right posterior gonopod, posterior-mesal view **G** left female vulva, posterior mesal view **H–L**SEM**H** left posterior gonopod, posterior-mesal view **I** tip of posterior gonopod, mesal view **J** apical part of posterior gonopod, mesal view **K** spiny lamellae near tip of posterior gonopod, mesal view **L** meso-distad process of posterior gonopod, posterior-mesal view. at = anterior gonopod telopodite; av = anterior valve; cx = coxa; pt = posterior gonopod telopodite; pv = posterior valve; st = sternum.

**Figure 4.**
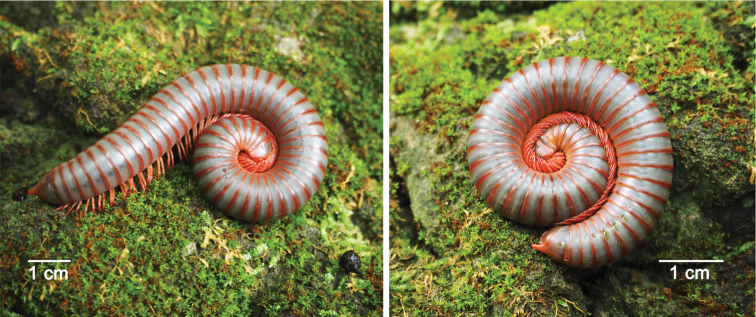
Live female *Macrurobolusmacrurus* comb. nov. from Wat Tham Inthanin, Thailand (CUMZ-D00147-3).

**Figure 5.**
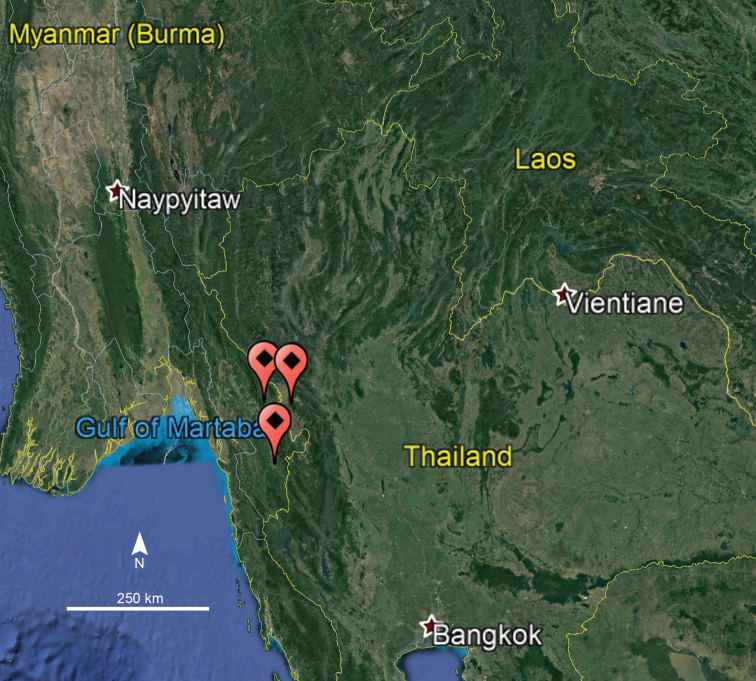
Distribution of *Macrurobolusmacrurus* comb. nov.

Figures 1 , 2 , 3 , 4 , 5

####### Diagnosis.

A genus of Pachybolidae characterised by the following combination of characters: preanal ring with long process protruding beyond anal valves; the anterior gonopod telopodite distally abruptly narrowed, forming an extremely long, slender, elevated process curved caudad.

####### Etymology.

The generic name is a combination of the name of the type species and “-bolus”, the ending of many pachybolid genus names.

####### Type species.

*Macrurobolusmacrurus* (Pocock, 1893) comb. nov.

*Spirobolusmacrurus*Pocock 1893: 396.

*Tonkinbolusmacrurus*: Hoffman 1962: 773.

*Atopochetusmacrurus*: Pimvichai et al. 2018: 174.

***Macrurobolusmacrurus***

####### (Pocock, 1893), comb. nov.

The original description was based exclusively on a female from “Kawkareet” (Tenasserim), Myanmar (see Distribution section for information on this locality). Pocock (1893) described the female external morphology and mentioned that this species differed from *Spiroboluscaudulanus* [= *Atopochetuscaudulanus* (Karsch, 1881)] and *Spirobolusmoulmeinensis* [= *Atopochetusmoulmeinensis* (Pocock, 1893)] by having a “much longer and thinner tail”.

####### Material studied.

Thailand, 1 ♂, 3 ♀♀; Tak Province, Mae Sot District, Wat Tham Inthanin; 16°45'59"N, 98°40'21"E; 660 m a.s.l.; 27 July 2016; P. Pimvichai, T. Backeljau and P. Prasankok leg. (CUMZ). • Myanmar, 1 ♂; Meetan; Fea; “ex typ.”; NHMD 621698.

####### Description of Thai specimens.

Adult male with 51 podous rings, no apodous rings. Length ca 11 cm, diameter ca 9.0 mm. Adult females with 48–51 podous rings, no apodous rings. Length ca 10–11 cm, diameter ca 10.0–10.4 mm.

Head capsule smooth, area below antennal sockets with wrinkles (Fig. 2A). Occipital furrow extending down between, but not beyond eyes; clypeal furrow reaching level of antennal sockets. Area below antennal sockets and eyes impressed, forming part of antennal furrow. Incisura lateralis open. 2+2 labral teeth, a row of labral setae, 1+1 supralabral setae (mentioned as “the labral region furnished with 4 punctures” by Pocock 1893: 401). Diameter of eyes ca half of interocular space; 9 vertical rows of ommatidia, 8 horizontal rows, 53–55 ommatidia per eye. Antennae short, not reaching beyond collum when stretched back, accommodated in a shallow furrow composed of a horizontal segment in the head capsule and a vertical segment in the mandibular cardo and stipes. Antennomere lengths 2 > 3 = 5 > 4 > 6 > 1 > 7; antennomere 1 glabrous, 2 and 3 with some ventral setae, 4, 5 and 6 densely setose; 4 apical sensilla. Mandibles: stipes (Mst) broad at base, apically gradually narrowed. Gnathochilarium (Fig. 2B): each stipes (Gst) with 3 apical setae; each lamella lingualis with 2 setae, one behind the other. Basal part of mentum (Me) transversely wrinkled; basal part of stipites longitudinally wrinkled.

Collum smooth, with a marginal furrow along lateral part of anterior margin; lateral lobes narrowly rounded, extending as far ventrad as the ventral margin of body ring 2.

Body rings 2–5 ventrally concave, hence with distinct ventrolateral “corners”. Body rings very smooth, parallel-sided in dorsal view. Prozona smooth. ‘Tergo-pleural’ suture visible on pro- and mesozona; mesozona ventrally with fine oblique striae, dorsally punctate; metazona ventrally with fine longitudinal striae, otherwise smooth. “Pleural” parts of rings with fine oblique striae. Sterna transversely striate. Ozopores from ring 6, situated in mesozona, ca 1/2 pore diameter in front of metazona (mentioned as “the repugnatorial pores situated in front of the transverse sulcus” by Pocock 1893: 401).

Telson smooth; preanal ring with slightly concave dorsal profile, with thick and long process protruding beyond anal valves (Fig. 2C). Anal valves (Av) impressed submarginally (Fig. 2D); margins hence distinctly protruding, liplike. Subanal scale (Sub) broadly triangular.

Legs (Fig. 2E): length of midbody legs 72–77% of body diameter in males, 54–56% of body diameter in females. Prefemur basally constricted, tarsus longer than other podomeres. First and second legs with 2 or 3 prefemoral, 2 or 3 femoral, 2 or 3 postfemoral, and 2–4 tibial setae, and 4 or 5 ventral and 1 dorsal apical setae on tarsi, numbers of setae reaching constancy from pair 3: each leg podomere from coxa to tibia with 1 seta; tarsi with 2 ventral apical and 1 dorsal apical seta, the apical ventral seta larger than the more basal one. Claw very slender, more than half as long as tarsus.

Colour. Living animal reddish brown except for grey pro- and mesozona (Fig. 4).

Male sexual characters. Tarsus from third to before the last 4 body rings with large ventral soft pad occupying entire ventral surface. Body ring 7 entirely fused ventrally, no trace of a suture. Tip of anterior gonopods visible when the animal is stretched out (not when it is rolled up).

Anterior gonopods (Fig. 3A, B, D, E) with triangular mesal sternal process, not reaching so far as the tip of coxae, apical margin bilobed, with basal longitudinal triangular ridge in posterior view. Coxa oval, apically gradually narrowed, rounded, projecting slightly beyond sternal process. Telopodite apically far overreaching coxa, distally abruptly narrowed, forming an extremely long, slender, elevated process curved caudad.

Posterior gonopods (Fig. 3C, F, H–I) strongly curved mesad, laterally with a massive ridge; with efferent canal (Enghoff 2011) running along mesal margin terminating in slender, pointed meso-distad process, covered with fine hairlike spinules (Fig. 3L); tip of posterior gonopod concave, apically ending in a rounded lobe (Fig. 3I, showed serrated margin, dorsally covered with short spines); with spiny lamellae mesally near tip.

Female vulvae (Fig. 3G). Valves prominent, of equal size; basally with open space between free margins.

####### DNA barcode.

The GenBank accession number of the COI barcode of the Thai specimen is MZ905519 (voucher code CUMZ-D00147).

####### Ecology

. Found under leaf litter.

####### Notes on the male from Meetan, Myanmar.

This specimen is labelled as “ex typ” in the NHMD collection and was, like the female type specimen, collected by Fea. It agrees with the Thai male in all characters, including all details of gonopod shape, with the following exceptions: Colour after > 100 years in alcohol is faded, but there is still a clear contrast between greyish pro- and mesozone and reddish-brown metazona. Size: length ca 8 cm, diameter 6.7 mm, 50 podous rings, no apodous rings in front of telson. Head capsule smooth. 11 vertical rows of ommatidia, of which 3 are very incomplete, 7 horizontal rows, 47 ommatidia per eye. Antennomeres 2–4 with some ventral setae, 5 and 6 densely setose. Gnathochilarium not dissected.

####### Distribution.

Tak Province, Thailand; Kawkareet (Tenasserim) and Meetan, Myanmar (Fig. 5). The names Kawkareet and Meetan do not appear on maps available to us. However, Brandis (2002: 1312) mentioned “Meetan (= Mitan Chaung (= river) 15°59'00"N 98°24'00"E at the south-west slope of the Dawna mountain”, whereas Randall and Page (2012: 344) located Meetan at “16.555556°N, 98.24°E (coordinates estimated)”. Annandale (1911: 118) stated that Kawkareet refers to Kawkareik and remarked in a footnote that “This locality [i.e. Kawkareik] is often referred to in zoological literature as Kawkareet or Kawkarit, or even Kokarit”. Finally, Likhitrakarn et al. (2017) located Kawkareet (= Kawkareik) at 16°33'20"N, 98°14'24"E and Meetan (= Mi Tan) at 16°00'12"N, 98°23'25"E.

## ﻿Discussion

The male specimen of *Spirobolusmacrurus* from Meetan in NHMD, although labelled “ex typ.”, should not a priori be regarded as a type (ICZN Art. 72.4.7.) because Pocock (1893: 396) explicitly mentioned that the species description was based on “A single ♀ from Kawkareet (Tenasserim)”. However, its non-sexual characters agree with Pocock’s (1893) description. Hence, we do not hesitate to refer it to *Macrurobolusmacrurus* comb. nov.

The new male specimen from Thailand and the old specimen from Myanmar share the long preanal ring process with the female type specimen, which is a remarkable character for a pachybolid, since most pachybolid genera (except *Aulacobolus* Pocock, 1903 and *Trachelomegalus* Silvestri, 1896) have a short preanal ring process. So, in this respect, *Macrurobolus* gen. nov. is clearly differentiated from most other pachybolid genera, including *Atopochetus* and *Litostrophus*, the two genera with which *Macrurobolus* gen. nov. appears the be most closely related in our phylogenetic tree (Fig. 1). Similarly, the anterior gonopod telopodites of *Macrurobolus* (telopodite distally abruptly narrowed, forming an extremely long, slender, elevated process curved caudad) clearly differ from those of *Litostrophus* (telopodite simple, without process, narrowly rounded) or *Atopochetus* (telopodite with a triangular process directed laterad originating on posterior surface at ~1/2 or 2/3–4/5 of its height). Hence, given that *Macrurobolus* shares neither the defining morphological synapomorphies of *Atopochetus*, nor those of *Litostrophus*, we think that the creation as a separate monotypic genus is warranted.

The interpretation of *Macrurobolus* as a separate genus is somehow in line with the COI tree (Fig. 1), which places the new genus in a clade comprising *Atopochetus* and *Litostrophus*, but which supports neither joining *M.macrurus* comb. nov. with *Atopochetus* (which itself forms a consistently well-supported clade), nor joining it with *Litostrophus* (which itself forms also a well-supported clade) (Fig. 1). Moreover, the mean interspecific COI sequence divergence between *M.macrurus* and other pachybolid and pseudospirobolellid species is 18% (range: 11–23%) (Tables 2, 3), a value that rather points to an intergeneric divergence (Table 3).

**Table 3.** Estimates of COI mean sequence divergences within (on diagonal) and among (below diagonal) pachybolid and pseudospirobolellid genera (range in parentheses) (data based on Pimvichai et al. 2018, 2020, 2022).

	**1**	**2**	**3**	**4**	**5**
1. *Apeuthes*	14 (11–16)				
2. *Atopochetus*	21 (18–23)	13 (8–16)			
3. *Coxobolellus*	19 (16–22)	22 (19–25)	12 (7–15)		
4. *Litostrophus*	19 (16–20)	16 (13–18)	20 (18–22)	11 (9–11)	
5. *Pseudospirobolellus*	21 (19–23)	22 (21–23)	21 (20–23)	22 (21–23)	14
6. *Macrurobolusmacrurus* comb. nov.	18 (18–21)	15 (14–17)	21 (20–23)	13 (11–14)	22 (21–23)

In conclusion, this study suggests that Pimvichai et al. (2018) appropriately labelled the transfer of *Tonkinbolusmacrurus* to the genus *Atopochetus* as “incertae sedis”. Indeed, the species can be accommodated in neither *Atopochetus* nor *Litostrophus*, i.e., the two genera with which it appears to be most closely associated. Hence, it would be ill-advised to maintain *Macrurobolusmacrurus* comb. nov. in the genus *Atopochetus*, for this would undermine both the definition and the support of the monophyly of this taxon. Therefore, the creation of the monotypic genus *Macrurobolus* gen. nov. seems the best solution to provide a generic name for *Spirobolusmacrurus* Pocock, 1893. Still, the monotypy of *Macrurobolus* gen. nov. renders it aphyletic *sensu*Ebach and Williams (2010), and hence in need of further study (Williams and Ebach 2020: 134).

## Supplementary Material

XML Treatment for Macrurobolus

## Supplementary materials

Supplementary material 1 Redescription of the giant SE Asian millipede *Spirobolusmacrurus* Pocock, 1893 and its assignment to the new genus *Macrurobolus* gen. nov. (Diplopoda, Spirobolida, Pachybolidae) Data type Jpg file. Explanation note Phylogenetic relationships of pachybolid and several other spirobolidan millipede species (excluding *Benoitolusbirgitae*) based on maximum likelihood analysis (ML) of a 660 bp COI gene fragment. https://binary.pensoft.net/file/649238This dataset is made available under the Open Database License (http://opendatacommons.org/licenses/odbl/1.0/). The Open Database License (ODbL) is a license agreement intended to allow users to freely share, modify, and use this Dataset while maintaining this same freedom for others, provided that the original source and author(s) are credited.Piyatida Pimvichai, Henrik Enghoff, Thierry Backeljau

Supplementary material 2 Table S2 Data type Xlsx file. Explanation note Estimates of COI sequence divergences (uncorrected *p*-distances) within and among Pachybolidae species and related taxa. https://binary.pensoft.net/file/649239This dataset is made available under the Open Database License (http://opendatacommons.org/licenses/odbl/1.0/). The Open Database License (ODbL) is a license agreement intended to allow users to freely share, modify, and use this Dataset while maintaining this same freedom for others, provided that the original source and author(s) are credited.Piyatida Pimvichai, Henrik Enghoff, Thierry Backeljau

## References

[B1] Annandale N (1911) The Fauna of Britih India, including Ceylon and Burma. Freshwater Sponges, Hydroids & Polyzoa. Taylor and Francis, London.

[B2] Brandis D (2002) On the taxonomic status and biogeography of the Isolapotamidae Bott, 1970 (Decapoda, Brachyura). Journal of Natural History 36: 1291–1339. 10.1080/00222930110051743

[B3] Ebach MC Williams DM (2010) Aphyly: a systematic designation for a taxonomic problem. Evolutionary Biology 37: 123–127. 10.1007/s11692-010-9084-5

[B4] Edgar RC (2004) MUSCLE: multiple sequence alignment with high accuracy and high throughput. Nucleic Acids Research 32: 1792–1797. 10.1093/nar/gkh340 15034147 PMC390337

[B5] Enghoff H (2011) East African giant millipedes of the tribe Pachybolini (Diplopoda, Spirobolida, Pachybolidae). Zootaxa 2753: 1–41. 10.11646/zootaxa.2753.1.1

[B6] Folmer O Black M Hoeh W Lutz R Vrijenhoek R (1994) DNA primers for amplification of mitochondrial cytochrome c oxidase subunit I from diverse metazoan invertebrates. Molecular Marine Biology and Biotechnology 3: 294–299. 7881515

[B7] Hebert PDN Cywinska A Ball SL DeWaard JR (2003) Biological identifications through DNA barcodes. Proceedings of the Royal Society London Series B 270: 313–321. 10.1098/rspb.2002.2218 PMC1691236 12614582

[B8] Hillis D Bull J (1993) An empirical test of bootstrapping as a method for assessing confidence in phylogenetic analysis. Systematic Biology 42: 182–192. 10.1093/sysbio/42.2.182

[B9] Hoffman RL (1962) Studies on spiroboloid millipeds IV. Systematic and nomenclatorial notes on the family Pachybolidae. Revue Suisse de Zoologie 69: 759–783. 10.5962/bhl.part.75592

[B10] Huelsenbeck JP Ronquist F (2001) MRBAYES: Bayesian inference of phylogeny. Bioinformatics 17: 754–755. 10.1093/bioinformatics/17.8.754 11524383

[B11] Kumar S Stecher G Li M Knyaz C Tamura K (2018) MEGA X: Molecular Evolutionary Genetics Analysis across computing platforms. Molecular Biology and Evolution 35: 1547–1549. 10.1093/molbev/msy096 29722887 PMC5967553

[B12] Lanfear R Frandsen PB Wright AM Senfeld T Calcott B (2017) PartitionFinder 2: new methods for selecting partitioned models of evolution for molecular and morphological phylogenetic analyses. Molecular Biology and Evolution 34: 772–773. 10.1093/molbev/msw260 28013191

[B13] Likhitrakarn N Jirapatrasilp P Golovatch S Panha S (2017) A checklist of the millipedes (Diplopoda) of Myanmar, with an updated list of Leonardo Fea’s collecting localities. Zootaxa 4350: 001–0046. 10.11646/zootaxa.4350.1.1 29245563

[B14] Miller MA Pfeiffer W Schwartz T (2010) Creating the CIPRES Science Gateway for inference of large phylogenetic trees. In: IEEE ‘Proceedings of the Gateway Computing Environments Workshop (GCE)’, 14 November 2010, New Orleans, LA, USA. INSPEC Accession Number: 11705685, 1–8. 10.1109/GCE.2010.5676129

[B15] Pimvichai P Enghoff H Panha S Backeljau T (2018) Morphological and mitochondrial DNA data reshuffle the taxonomy of the genera *Atopochetus* Attems, *Litostrophus* Chamberlin and *Tonkinbolus* Verhoeff (Diplopoda: Spirobolida: Pachybolidae), with descriptions of nine new species. Invertebrate Systematics 32: 159–195. 10.1071/IS17052

[B16] Pimvichai P Enghoff H Panha S Backeljau T (2020) Integrative taxonomy of the new millipede genus *Coxobolellus*, gen. nov. (Diplopoda: Spirobolida: Pseudospirobolellidae), with descriptions of ten new species. Invertebrate Systematics 34(6): 591–617. 10.1071/IS20031

[B17] Pimvichai P Panha S Backeljau T (2022) Combining mitochondrial DNA and morphological data to delineate four new millipede species and provisional assignment to the genus *Apeuthes* Hoffman & Keeton (Diplopoda: Spirobolida: Pachybolidae: Trigoniulinae). Invertebrate Systematics 36(2): 91–112. 10.1071/IS21038

[B18] Pocock RI (1893) Viaggio di Leonardo Fea in Birmania e regioni vicine LV. On the Myriopoda of Burma. Pt 3. Report upon the Julidae, Chordeumidae and Polyzonidae collected by Sig. L. Fea and Mr. E.W. Oates. Annali del Museo civico di Storia naturale di Genova 33: 386–406.

[B19] Randall ZS Page LM (2012) Resurrection of the genus *Homalopteroides* (Teleostei: Balitoridae) with a redescription of *H.modestus* (Vinciguerra 1890). Zootaxa 3586: 329–346. 10.11646/zootaxa.3586.1.31

[B20] San Mauro D Agorreta A (2010) Molecular systematics: a synthesis of the common methods and the state of knowledge. Cellular & Molecular Biology Letters 15: 311–341. 10.2478/s11658-010-0010-8 20213503 PMC6275913

[B21] Stamatakis A (2014) RAxML version 8: a tool for phylogenetic analysis and post-analysis of large phylogenies. Bioinformatics 30: 1312–1313. 10.1093/bioinformatics/btu033 24451623 PMC3998144

[B22] Williams DM Ebach MC (2020) Cladistics – a Guide to Biological Classification (3^rd^ edn.). Systematics Association Special Volume 88, Cambridge University Press, UK.

[B23] Xia X (2018) DAMBE7: new and improved tools for data analysis in molecular biology and evolution. Molecular Biology and Evolution 35: 1550–1552. 10.1093/molbev/msy073 29669107 PMC5967572

