# Supplementary figures and images for: ﻿Redescription of the giant Southeast Asian millipede Spirobolusmacrurus Pocock, 1893 and its assignment to the new genus Macrurobolus gen. nov. (Diplopoda, Spirobolida, Pachybolidae)
Source: Zookeys. 2022 Feb 22;1087:1–18. doi: 10.3897/zookeys.1087.71280 (PMC8888539; doi:10.3897/zookeys.1087.71280)

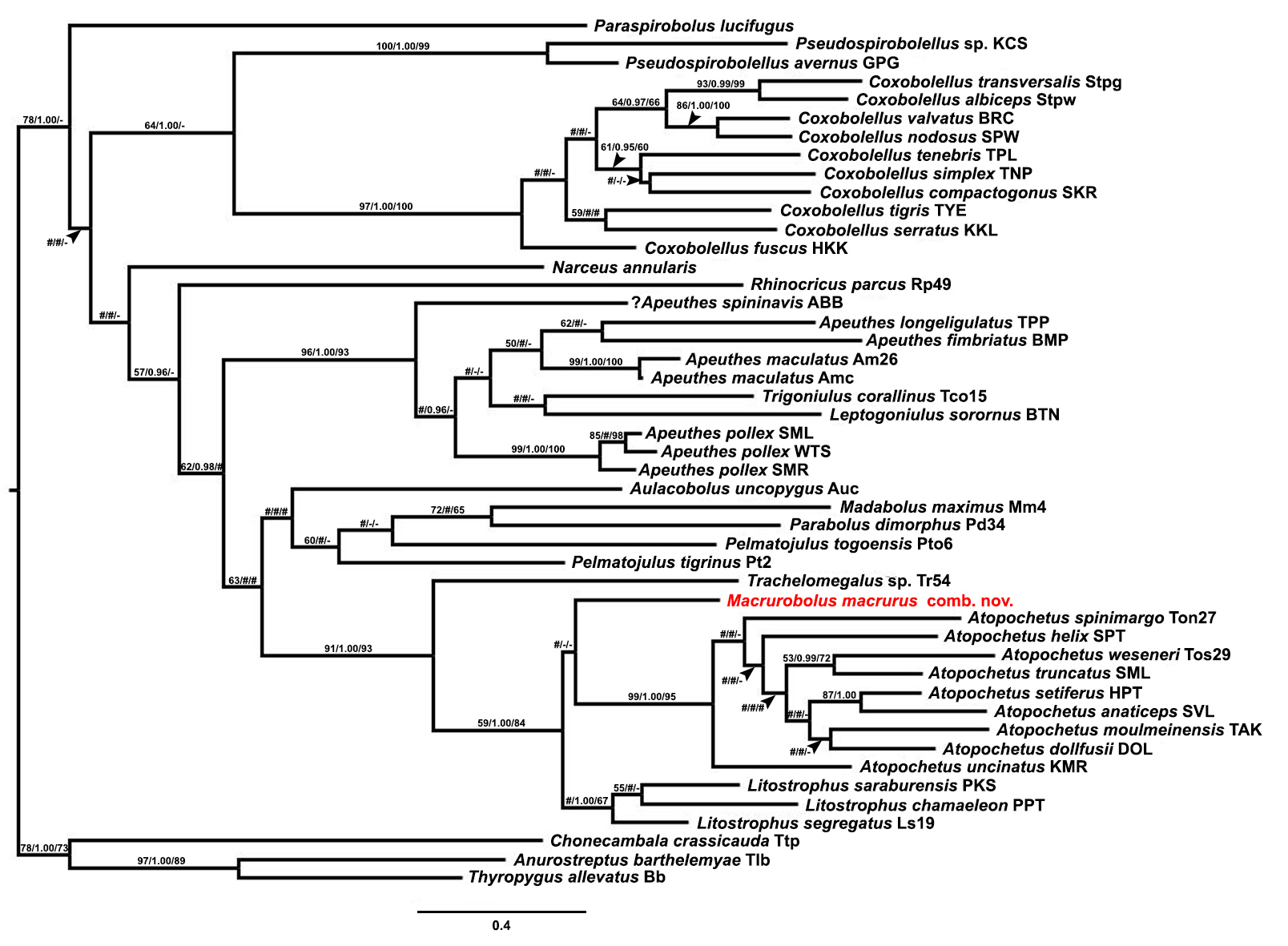

Supplement: Supplementary material 1 — Redescription of the giant SE Asian millipede Spirobolusmacrurus Pocock, 1893 and its assignment to the new genus Macrurobolus gen. nov. (Diplopoda, Spirobolida, Pachybolidae) [file zookeys-1087-001-s001.jpg]
